# The Influence of Graded Amount of Potassium Permanganate on Corrosion of Hot-Dip Galvanized Steel in Simulated Concrete Pore Solutions

**DOI:** 10.3390/ma15217864

**Published:** 2022-11-07

**Authors:** Petr Pokorný, Vítězslav Vacek, Nikola Prodanovic, Adam Zabloudil, Jaroslav Fojt, Viktor Johánek

**Affiliations:** 1 Department of Building Materials, Klokner Institute, Czech Technical University in Prague, 166 08 Prague, Czech Republic; 2 Department of Metals and Corrosion Engineering, University of Chemistry and Technology Prague, 166 28 Prague, Czech Republic; 3 Department of Surface and Plasma Science, Faculty of Mathematics and Physics, Charles University, 121 16 Prague, Czech Republic

**Keywords:** corrosion, concrete, hot-dip galvanized steel, reinforcement, permanganate, hydrogen evolution

## Abstract

This paper evaluates the amount of KMnO_4_ in simulated concrete pore solution (pH 12.8) on the corrosion behaviour of hot-dip galvanized steel (HDG). In the range of used MnO_4_^−^ (10^−4^, 10^−3^, 10^−2^ mol·L^−1^), corrosion behaviour is examined with regard to hydrogen evolution and composition (protective barrier properties) of forming corrosion products. The corrosion behaviour of HDG samples is evaluated using R_p_/E_corr_ and EIS. The composition of corrosion products is evaluated using SEM, XRD, XPS and AAS. The effective MnO_4_^−^ ion concentration to prevent the corrosion of coating with hydrogen evolution is 10^−3^ mol·L^−1^; lower concentrations only prolong the time to passivation (corrosion with hydrogen evolution). The highest used MnO_4_^−^ concentration ensures corrosion behaviour without hydrogen evolution but also leads to the formation of less-protective amorphous corrosion products rich in Mn^II^/Mn^III^ phases.

## 1. Introduction

The use of coatings in the protection of conventional concrete reinforcement can provide effective means of prolonging the service life of reinforced structures [1,2]. The rate of production and the relatively low cost also predetermine the use of hot-dip galvanized coating [1,3,4]. The results of research evaluating the use of hot-dip galvanized steel for concrete reinforcement demonstrated that the coating was resistant to the carbonation of cover layer and showed increased resistance against chloride anions [5,6,7,8]. On the other hand, it is obvious that HDG steel corrodes at an unacceptable rate in fresh and curing concrete (see anodic reaction—Equation (1)) with hydrogen evolution (cathodic reation—Equation (2)) [9,10,11].

(1) $$\mathrm{Zn}\to {\mathrm{Zn}}^{2+}+2e^{-}\;(E\;\sim -960\;\mathrm{mV}/\mathrm{ACLE})$$

(2) $$2H_{2}O+2e^{-}\to H_{2}+2{\mathrm{OH}}^{-}\;(E\;\sim -939\;\mathrm{mV}/\mathrm{ACLE},\;\mathrm{at}\;\mathrm{pH}\;12.8)\;$$

These initial concurrent corrosion reactions significantly limit the use of hot-dip galvanized rebar for concrete reinforcement. Anodic corrosion reaction reduces the coating thickness, and cathodic reaction (hydrogen evolution) increases the porosity of the cement paste at phase interface. This increase in porosity can reduce the bond strength between coated rebar and concrete [9,12,13,14].

Some authors suggest that both effects can be hindered in later stages by the formation of zinc corrosion product: Ca[Zn(OH)_3_]_2_·2H_2_O (calcium hydroxyzincate—CHZ) [15,16,17,18,19]. Others show that the effect is insufficient [20,21].

The conclusions of some experimental works show positive effects of zinc corrosion products on filling pores formed due to hydrogen evolution, ultimately leading to increased bond strength [22,23,24]. Other authors found the bond strength to be reduced [9,20,25,26,27].

If RDHE (rapid dissolution and hydrogen evolution) can be limited, the use of hot-dip galvanized coating will be feasible, from the point of both the sufficient strength of the structure and the prolongation of service life. The use of additional coating can reduce RDHE significantly. With this goal, the use of chromate conversion coatings has been studied [28,29,30]. Coating with Cr^VI^ (e.g., yellow chromate conversion coating) is no longer feasible due to legislative limitations (toxicity) despite very good protective ability [31,32]. Cr^III^ conversion coatings are significantly less effective compared with Cr^VI^-enriched conversion coatings [33]. Some studies admit positive effects of other conversion coatings on RDHE. Among these are phosphate [34] and, alternatively, Ce or Ce/La coatings [35,36]. Older literature sources discuss the high effectivity of bitumen-based coatings [37,38,39].

Another method of RDHE reduction is the use of suitable corrosion inhibitors in cement paste. In the 1960s, water-soluble CrO_4_^2−^ (100–200 ppm) was shown to reduce hydrogen formation during hot-dip galvanized coating corrosion [40]. Since the natural amount of CrO_4_^2−^ in current cement production is very low (<10 ppm), the concentration needs to be increased [41]. While the positive effect of chromate anions on RDHE reduction has been proven [42,43,44], it is no longer feasible due to legislative limitations ([31,32]).

Other compounds have been tested with similar goals. The addition of H_2_O_2_ significantly reduces RDHE, but forming oxygen again increases the porosity of cement paste (reduces concrete mechanical properties) [45]. Sodium eperuate added to cement increases the corrosion resistance of hot-dip galvanized steel in concrete, but it has not been proven that it inhibits the hydrogen formation [46]. The effective limitation of the cathodic corrosion reaction of hot-dip galvanized steel in concrete with hydrogen evolution was achieved (addition of 0.005 wt. % of mixing water) by N-trimethylsylilimidazole, benzimidazole and derivates benzimidazole (e.g., 2-merkaptobenzimidazolu). It was also verified that these compounds do not have, in the amounts used, effects on cement mechanical properties [47]; however, considering their toxicity, they cannot be used in civil engineering constructions [48,49].

Recently, the effect of KMnO_4_ in simulated concrete pore solution on the corrosion behaviour of hot-dip galvanized steel was studied [50]. According to the results, the presence of 10^−3^ mol·L^−1^ of MnO_4_^−^ significantly reduces RDHE and therefore stabilizes the bond strength of concrete and reinforcement as well as the barrier effect of the coating. While it has been shown that the hydration process of conventional cement is not affected by KMnO_4_ addition [51], the use of this compound is strictly limited due to its ecotoxicity [52,53]. For this reason, it is necessary to verify the effects of different concentrations on the corrosion process and based on the results, assess their feasibility for civil engineering constructions. A study of KMnO_4_ in simulated concrete pore solution also allows for studying in detail the inhibition mechanism and coating composition.

This paper studies the effect of MnO_4_^−^ on the corrosion inhibition of HDG steel in simulated concrete pore solutions. It focuses on the electrochemical process and on the changes in the composition of corrosion products (relative to increasing amounts of MnO_4_^−^ in simulated concrete pore solution). The article cited above [50] does not describe the formation of coating in detail; however, it uses XPS to show that the coating includes calcium hydrozincite (CHZ), which is expected, and corrosion products based on Ca and Mn (CaMnO_4_, Ca(HMnO_2_)_2_) as well as phases based solely on Mn: MnO_2_, Mn_2_O_3_, Mn_3_O_4_, MnO, MnO(OH) and even Mn. This corrosion products showed a protective effect of coating comprising both Ca[Zn(OH)_3_]_2_·2H_2_O and compounds with Mn in different oxidation states.

## 2. Materials and Methods

For the sample production, flat carbon steel sheets of 40 mm width were used. The composition is shown in Table 1 (producer-guaranteed values). Steel was first hot-dip galvanized (wet galvanizing process, Apollo Metal, Ltd. establishment Čenkov, Czech Republic, conventional hot-dip galvanizing according to the hot-dip galvanizing establishment regulations) and then sectioned using automatic saw MTH mikron 3000 to a final size of 40 × 40 mm^2^. Prior to hot-dip galvanizing, the samples were (in laboratory ambient condition, i.e., 22 ± 1 °C, standard pressure) degreased in 10 wt. % NaOH and pickled in 12 wt. % HCl solution. After pickling, the samples were rinsed in multiple steps (pressure washing with water, dipping in distilled water). During the hot-dip galvanizing process in the automatic coating line, the samples were again pretreated in alkaline degreasing solution (8–10 wt. % KOH in destiled water, 60 °C, exposition time: 10 min) and pickled in 7–12 wt. % HCl solution (20 ± 1 °C, exposition time: 5 min). After coating, the samples were cooled in ambient air.

In the first step, cross cuts through coating were prepared, and the cross-section microstructure of the Fe-Zn alloy coating was documented using light microscopy (Nicon Eclipse 600, Nikon Corporation, Tokyo, Japan). Cross sections were prepared using automatic grinder/plisher LaboPol—2, Struers LLC, (Cleveland, OH, USA) and emery papers P60-P2400 (Metalco Material Testing, Ltd., Roztoky, Czech Republic) and diamond polishing pastes (grain size: 3 µm, 1 µm and 0.25 µm, Metalco Material Testing, Ltd., Roztoky, Czech Republic). The cross sections were not leached. Exposure solutions (simulated concrete pore solutions) with step-wise controlled concentrations of KMnO_4_ were produced (Table 2). Calcium oxide was dissolved in distilled water, forming a saturated solution with pH 12.60–12.65 (pH eletrode JENWAY 3540, UK, stabilisation 20 min, 21 ± 1 °C). Then, KOH was added in amount to reach pH 12.80. The simulated concrete pore solutions thus realized were created according to the model given in [54,55]. KOH was deliberately used to increase the pH of the simulated concrete pore solution because the use of NaOH increases the pH measurement error of alkaline solutions due to the so-called natural sodium error [56,57,58,59]. In addition, the solubility of KOH is higher than NaOH, and this speeds up the preparation of solutions [60,61,62]. Further, KMnO_4_ was added, except for the reference solution (see Table 2). Concentration of MnO_4_^−^ in simulated concrete pore solution of 10^−3^ mol·L^−1^ is referenced as suitable for suppressing RDHE (solution denominated Mn^(2)^) [50]. In our experiment, we chose concentrations one order of magnitude lower (solution denominated Mn^(1)^—10^−4^ mol·L^−1^) and one order higher (Mn^(3)^—10^−2^ mol·L^−1^). The chosen concentration of KMnO_4_ (10^−4^ mol·L^−1^) in the model concrete pore solution, which is an order of magnitude lower than the standard (10^−3^ mol·L^−1^ [50]), represents a significant under-dosing of this substance in real building mixtures. Conversely, an order of magnitude higher concentration of KMnO_4_ (10^−2^ mol·L^−1^) than the standard indicates a substantial overdosing (mainly local non-mixing) of this substance in construction mixtures. Testing the corrosion behaviour of hot-dip galvanized steel in a model concrete pore solution with MnO_4_^−^ added in a wide concentration range is important for verifying the behaviour under realistic conditions in the construction industry. In fact, it is often difficult to ensure the dosing of sufficient amounts of inhibitors into concrete mixtures under realistic conditions of large concrete structures.

The exposure was started immediately after simulated concrete pore solution preparation, and the total duration of exposure was 2 months. Exposure vessels were covered with PE foil to prevent entry of O_2_/CO_2_ from the atmosphere.

After exposure, the samples were rinsed by distilled water and dried in the ambient atmosphere (72 h, 65 % RH, 21 ± 1 °C). Composition and morphology of corrosion products on the sample surface were then analyzed. Morphology and thickness were evaluated using SEM (Zeiss—Auriga Compact, Carl Zeiss AG, Jena, Germany). Composition of corrosion products was analyzed with X-ray diffraction (Malvern Panalytical V. B., Almelo, The Netherlands) using CuKα radiation over the angular range 5–90° and XPS (Phoibos MDC 9 electron energy analyzer, SPECS GmbH, Berlin, Germany). All XPS spectra were measured at primary photon energy hν = 1486.6 eV (non-monochromatized Al Kα radiation) with detection at normal emission angle by MDC 9 electron energy analyzer. Apart from a wide survey spectrum, detailed spectral regions of Mn 2p, Mn 3s, Zn 2p, Zn 3p, O 1s and C 1s were acquired, along with the Auger series of Mn LMM and Zn LMM. Surface charging was removed using the C 1s (sp^2^ carbon) line position in the spectra. Shirley method was applied for background subtraction in all cases. Expected penetration was 2–3 nm. SEM sampling (same device; see above) was also used for detailed analysis of cross sections on samples after exposure (determination of thickness and composition of corrosion products—element maps). Metallographical cuts were made identically to the method for reference samples.

Further, corrosion products were removed using 15 wt. % HCl for 15 min. The goal was to determine the ratio between manganese and calcium via analysis using device GBC932+ (GBC Scientific Equipment Ltd., Dandenong, Australia).

Electrochemical corrosion tests included measurement of corrosion potential (E_corr_) and polarization resistance (R_p_) trend for the duration of 162 h. In both cases, four parallel samples were measured at every hour. Reference was saturated silver–silver chloride electrode (SSCE). After assembly of the setup and filling of cells with simulated concrete pore solution, the start of measurement was delayed 15 min before starting the sequential measurement E_corr_ and R_P_ (so called R_p_/E_corr_ trend). Total exposed area of the samples was 4.5 cm^2^. Polarization resistance value was evaluated as the linear coefficient of current density and potential in the vicinity of E_corr_ (−20 to +20 mV). Rate of polarization was 0.1 mV·s^−1^. The electrochemical impedance spectra were measured in the frequency range from 60 kHz to 10 mHz with 10 mV/E_corr_ AC amplitude within a 14 h period. The measurement was done in a three-electrode setup in a PTFE press-on cell of cylindrical shape (total area of sample was always 4.5 cm^2^). Glassy carbon rods were used as counter-electrodes placed in the axis of capillary outlet. Electrochemical measurements were done using Electrochemical Multiplexer (allows measurement of 4 parallel samples), ECM 8 with potentiostat Reference 600 (Gamry Instruments, Warminster, PA, USA).

**Figure 1:**
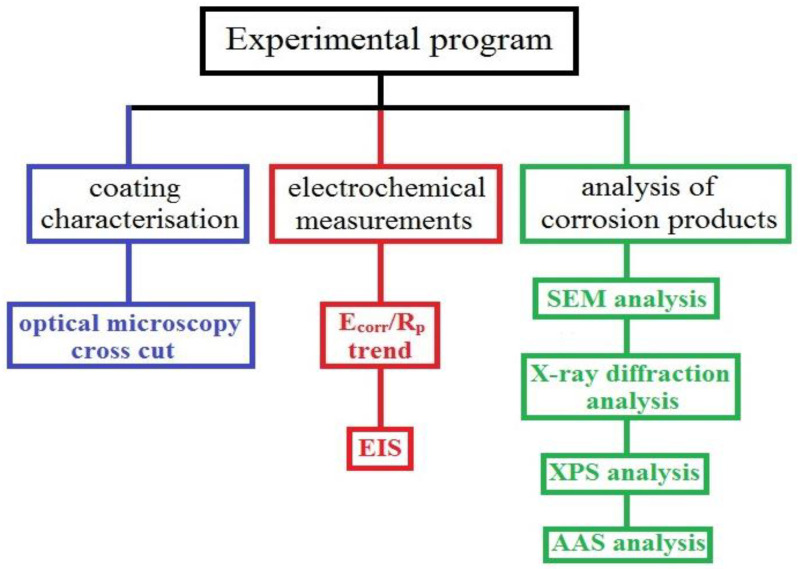
Flow chart of experimental program of this study.

The experimental program of this work includes both electrochemical measurements and tests evaluating HDG coating composition and corrosion products. For illustrative purposes, a flow chart of the experimental program of this study is shown in Figure 1.

## 3. Results and Discussion

### 3.1. Structure of HDG Coating

**Figure 2:**
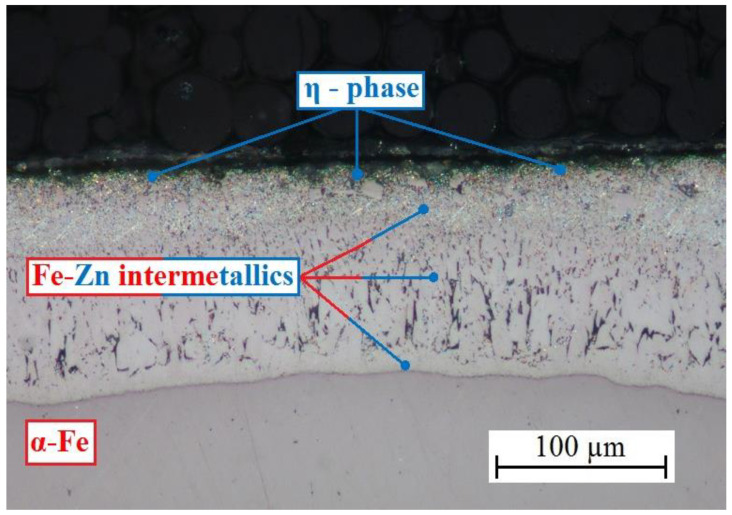
Cross cut of HDG zinc coating on flat steel sheets—overview.

**Figure 3:**
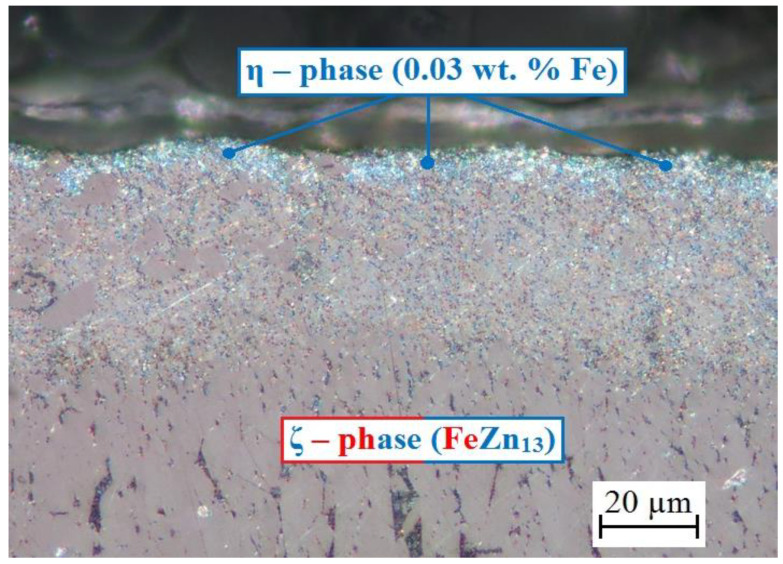
Cross cut of HDG zinc coating on flat steel sheets—detail view of the outer layer.

Images of cross-cut structure of the hot-dip galvanized coating are shown in Figure 2 and Figure 3. A cross cut through the whole coating of average thickness 100 µm is shown in Figure 2 and Figure 3 shows a detailed view of the outer part of the coating. Used galvanized steel with silicon content of 0.18–0.22 wt. % falls into Sebisty range (0.15–0.25 wt. %) [63,64], ensuring formation of relief structure on inner surface of the intermetallic Fe-Zn phases (Γ group (Γ + Γ_1_), δ group (δ_1p_ + δ_1k_) and phase ζ) and outer layer comprising of η phase (see Figure 2) [65,66,67,68].

If the silicon content was in Sandelines range (0.03–0.12 wt. %), the coating would comprise from 90% of brittle ζ phase (FeZn_13_) [63,69,70]. Phosphorus content has no effect on composition of coating [68,71,72]. Outer part of the coating (Figure 3) includes evidently uniform layer of η phase (solid solution of iron in zinc, iron content is cca 0.03 wt. %) of 10–15 µm thickness. Only under this phase can the palisade-like structure of ζ phase (FeZn_13_) be found. It is possible that η phase contains some isolated crystallites of ζ phase, however the images clearly show prevalence of η phase. Its thickness (at least 10 µm) is, based on available literature, sufficient to passivate zinc (formation of CHZ layer) at the pH of simulated concrete pore solutions (presence of Ca(OH)_2_) < 13.3 ± 0.1 [73,74,75,76]. It has been proven that intermetallic Fe-Zn phases generally exhibit lower corrosion resistance in simulated concrete pore solutions compared to pure zinc or η phase [76,77,78,79].

It is apparent from the cross cuts that the coating has standard composition with uniform outer layer of η phase of sufficient thickness (ensuring easy passivation of simulated concrete pore solutions of pH up to 13.3). Visual evaluation of coated samples did not reveal any coating degradation such as ash, rundown, or scaling [80].

### 3.2. Electrochemical Measurements

#### 3.2.1. E_corr_/R_p_ Trend

**Figure 4:**
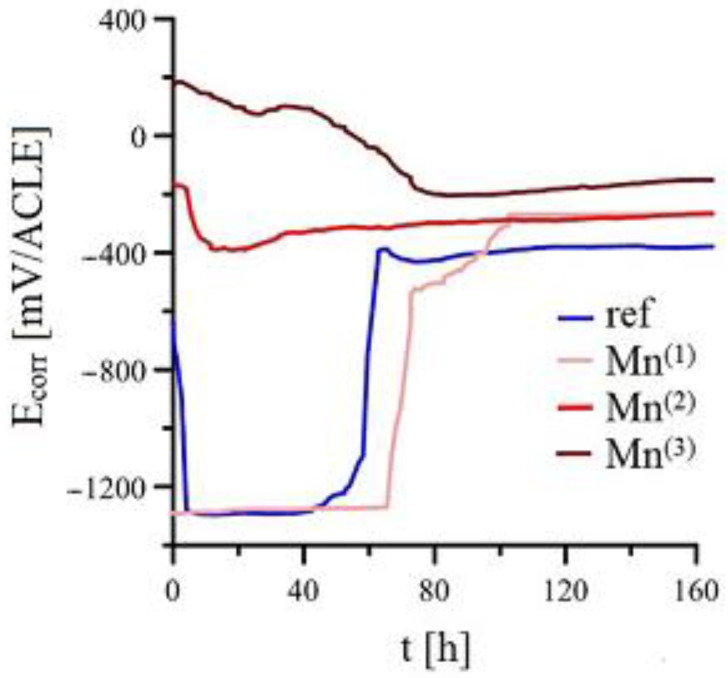
Time dependency of E_corr_ for 4 parallel samples in simulated concrete pore solutions.

**Figure 5:**
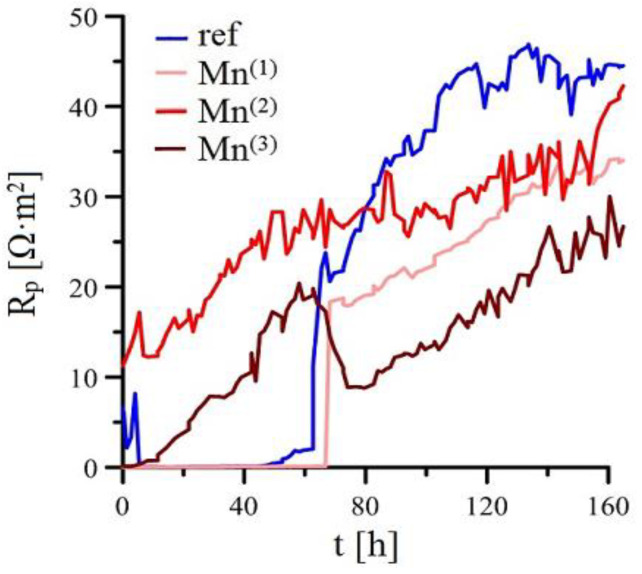
Time dependency of R_P_ for 4 parallel samples in simulated concrete pore solutions.

Figure 4 shows the recorded time dependence of free corrosion potential (E_corr_) of HDG samples in simulated concrete pore solutions with KMnO_4_ (Mn^(1)^–Mn^(3)^), and Figure 5 shows the time dependence of Polarization resistance (R_p_/Ω·m^2^) in the same experimental setup. In both cases, the curves are produced as an average of four measurements. Individual curves showed statistically insignificant differences.

The R_p_/E_corr_ trend for reference samples is similar to that found in literature [2,7,9,21,33,39,73,74,75,76]. Initially, the sample of hot-dip galvanized coating corrodes with hydrogen evolution (1) at roughly −1200 mV/ACLE and very low values of polarization resistance (in the order of tenths of Ω·m^2^). After a while (~50 h), E_corr_ is increased (~−400/ACLE) and simultaneously the R_p_ (increase to 35–45 Ω·m^2^). A layer of corrosion products is formed, primarily composed of Ca[Zn(OH)_3_]_2_·2H_2_O which strongly inhibits the corrosion damage to the HDG steel in this environment [9,21,73,74,75,76,81,82,83,84]. It is a matter of discussion if passivation of HDG samples is also affected by formation of ZnO/Zn(OH)_2_ by filling the pores between Ca[Zn(OH)_3_]_2_·2H_2_O) [9,21].

R_p_/E_corr_ trend of HDG samples in simulated concrete pore solution (pH 12.8) with addition of only 10^−4^ mol·L^−1^ KMnO_4_ (Mn^(1)^) is very similar to the trend in solution without any KMnO_4_ (ref). Nevertheless, the time to the passivation of HDG samples is slightly (several hours) increased. The total time of coating corrosion with hydrogen evolution is similar to or even longer than that for samples exposed in simulated concrete pore solution without any addition of KMnO_4_. The effect of MnO_4_^−^ on precipitation of common corrosion products (CHZ, ZnO, Zn(OH)_2_) is not apparent from E_corr_ trend; however, R_p_ is about 20 % lower past 120 h compared to reference solution, suggesting that the corrosion products might be altered. It is feasible that this solution allows competing cathodic reactions of reduction MnO_4_^−^ or MnO_4_^(−2)^ to, for example, MnO_2_. The reduction of MnO_4_^−^ to MnO_4_^(−2)^ in an alkaline environment (3) proceeds relatively quickly [85,86]; the eventual reduction of MnO_4_^−^ to MnO_2_ is described by Equation (4) [86,87]. Manganese can be reduced from Mn^IV^ (MnO_2_) to Mn^III^ (Mn_2_O_3_, Equation (5)) [88], or even Mn^II/III^ (Mn_3_O_4_, Equation (6)) [88]. The reduction of manganese from MnO_2_ can continue until MnO(OH) is formed—see Equation (7) [89]. Potentials were calculated based on equilibria from E-pH diagrams (pH = 12.8, t = 25 °C) [88].

(3) $${\mathrm{MnO}}_{4}^{-}+e^{-}\to {\mathrm{MnO}}_{4}^{\left(-2\right)}\left(E\;\sim +329\;\mathrm{mV}/\mathrm{ACLE}\right)$$

(4) $${\mathrm{MnO}}_{4}^{-}+2H_{2}O+3e^{-}\to {\mathrm{MnO}}_{2}+4{\mathrm{OH}}^{-}\left(E\;\sim +353\;\mathrm{mV}/\mathrm{ACLE}\right)$$

(5) $$2{\mathrm{MnO}}_{2}+H_{2}O+2e^{-}\to {\mathrm{Mn}}_{2}O_{3}+2{\mathrm{OH}}^{-}\left(E\;\sim +61\;\mathrm{mV}/\mathrm{ACLE}\right)$$

(6) $$3{\mathrm{Mn}}_{2}O_{3}+H_{2}O+2e^{-}\to 2{\mathrm{Mn}}_{3}O_{4}+2{\mathrm{OH}}^{-}\left(E\;\sim -264\;\mathrm{mV}/\mathrm{ACLE}\right)$$

(7) $${\mathrm{MnO}}_{2}+H_{2}O+e^{-}\to \mathrm{MnO}\left(\mathrm{OH}\right)+{\mathrm{OH}}^{-}\left(E\;\sim -7\;\mathrm{mV}/\mathrm{ACLE}\right)$$

In case of exposure of HDG in simulated concrete pore solution at pH 12.8 with the addition of 10^−3^ mol·L^−1^ KMnO_4_ (Mn^(2)^), significant change of R_p_/E_corr_ can be observed. The time dependence of E_corr_ suggests coating corrosion without hydrogen evolution (E_corr_ is initially around −200–−400 mV/ACLE) according to Equation (2). It is apparent that this content of MnO_4_^−^ is sufficient to completely prevent the corrosion of coating with hydrogen. The coating corrodes with the reduction of atmospheric oxygen (Equation (8)) [50,90]. The above stated range of E_corr_ can also facilitate other, competing cathodic reactions with manganese (Equation (9)) [91,92].

(8) $$O_{2}+2H_{2}O+4e^{-}\to 4{\mathrm{OH}}^{-}\left(E\;\sim +201\;\mathrm{mV}/\mathrm{ACLE}\right)$$

(9) $$\mathrm{Mn}{\left(\mathrm{OH}\right)}_{3}+e^{-}\to \mathrm{Mn}{\left(\mathrm{OH}\right)}_{2}+{\mathrm{OH}}^{-}\left(E\;\sim -47\;\mathrm{mV}/\mathrm{ACLE}\right)$$

Since the beginning of the exposure, R_p_ values an order of magnitude higher were recorded compared the exposures in reference solution. This can be explained by the decrease in oxygen transport towards the coating but inhibition of the anodic reaction itself (see (1)), as discussed by previous authors [50]. From the long-term point of view (R_p_ past 120 h), it is apparent that forming corrosion products (probably Mn-rich phases in different oxidation state), HDG gives the samples lower protective barrier properties (reduction of 20%) compared the common corrosion products (see R_p_ after 120 h for reference samples). From this point of view it can be argued that presence of common HDG corrosion products up until pH 13.3 (CHZ, ZnO/Zn(OH)_2_) [9,14,76,81,82,84] provides the coating with higher barrier properties compared to exposure in environments with MnO_4_^−^ sufficient to considerably reduce RDHE.

Initial values of E_corr_ of HDG samples in simulated concrete pore solution with 10^−2^ mol·L^−1^ KMnO_4_ (Mn^(3)^) are reached values as high as +150–+200 mV/ACLE. Long term evolution of E_corr_ (>60 h) is similar to that in the Mn^(2)^ solution. Evolution of E_corr_ suggests that at this concentration of KMnO_4_ in simulated concrete pore solution (pH 12.8), hydrogen evolution is prevented (reaction (2)). Although compared to exposure in solution Mn^(2)^ the increase of Rp is gradual and initially measured values (tenths of Ω·m^2^) are similar to exposure in simulated concrete pore solution without any KMnO_4_. Therefore the anodic reaction rate is increased (reaction 1) and the solution also contains sufficient amounts of oxygen to compensate (see (8)). It can be expected that composition of corrosion products differs from the reference (ref) exposure, the presence of Mn-rich products is likely. These products, compared to common zinc corrosion products (CHZ, ZnO/Zn(OH)_2_) will, after longer exposure, show worse barrier properties (45 % lower R_p_ at the end of exposure compared to HDG in reference exposure).

**Figure 6:**
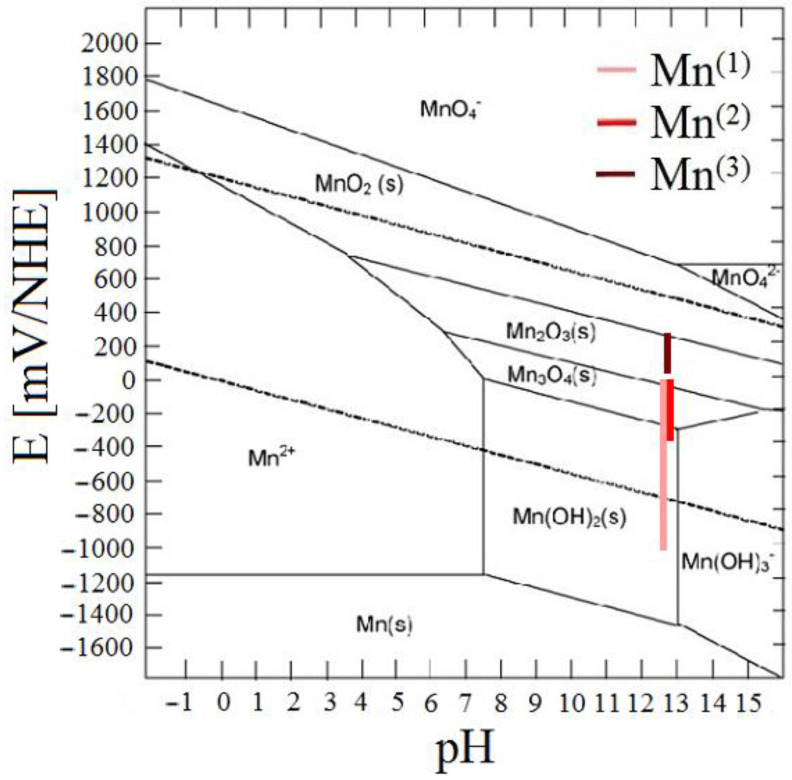
E-pH (equilibrium) diagram of manganese, marked areas signify stability ranges for individual phases E_corr_ of HDG in simulated concrete pore solutions with KMnO_4_ [88].

Figure 6 shows potential ranges (pH = 12.8, t = 25 °C) of existence as function of time and E_corr_ of HDG samples in simulated concrete pore solution with KMnO_4_. In the case of the potential stability of individual phases, Mn(OH)_2_ transforms to Mn_3_O_4_ (Mn^II/III^), in case of Mn^(2)^ exposure, the potential is in the range of stability of Mn_3_O_4_. On the contrary, exposure Mn^(3)^ showed E_corr_ of HDG essentially only in the range of Mn_2_O_3_ (Mn^III^) stability.

#### 3.2.2. EIS

**Figure 7:**
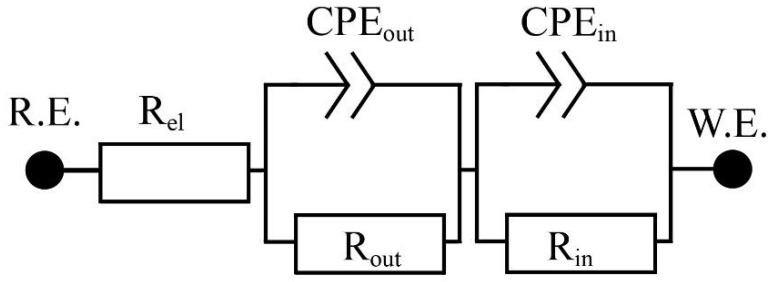
Equivalent circuit used to evaluate EIS spectra.

To provide a more fundamental understanding of the electrochemical mechanism of corrosion of HDG in simulated concrete pore solutions with KMnO_4_, EIS (electrochemical impedance spectroscopy) were measured and evaluated. Equivalent circuit model used for evaluation is in Figure 7, including CPE_out_ and R_out_ (capacity and resistance of layer of corrosion layer precipitate) CPE_in_ a R_in_ (capacity and resistance of charge transfer) and R_el_ for electrolyte resistance. The equivalent circuit was chosen based on the shape of the spectrum and the expected corrosion mechanism. The other considered was Rs (CPE-R(CPE-R)), however, our chosen circuit provided a higher quality fit. A CPE (constant phase element) is used in EC as a substitute for capacitors, taking into account the non-ideal behavior of the system. This element is defined as Z = [C·(jω)α] − 1, where α takes values in range 0 to 1. Values close to 0 correspond to the behavior of the resistor and values close to 1 to the capacitor.

**Figure 8:**
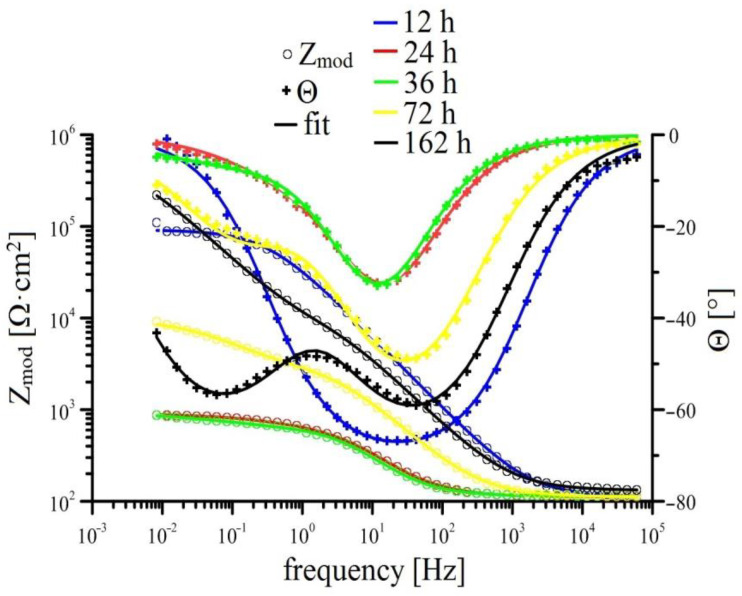
Time-dependent impedance spectra for reference solution (Bode plot: log Z_mod_ vs. log frequency and phase angle vs. log frequency).

**Figure 9:**
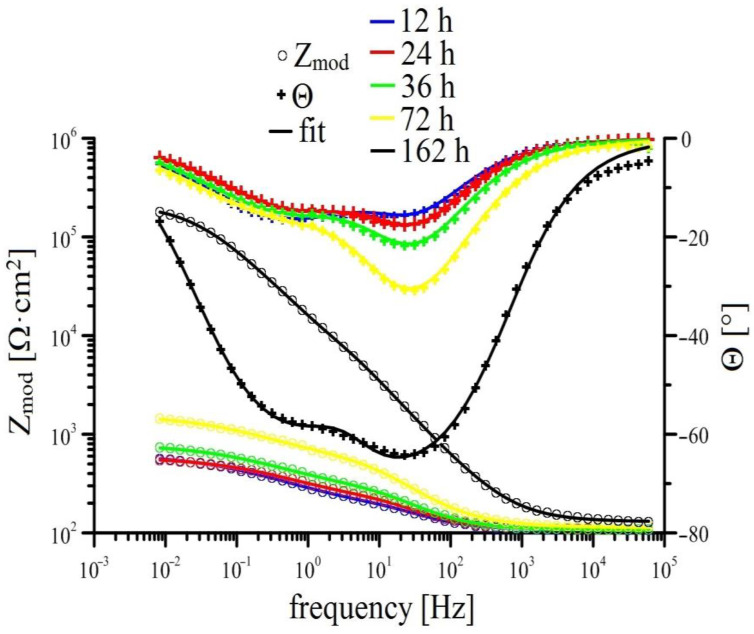
Time-dependent impedance spectra for Mn^(1)^ solution (Bode plot: log Z_mod_ vs. log frequency and phase angle vs. log frequency).

**Figure 10:**
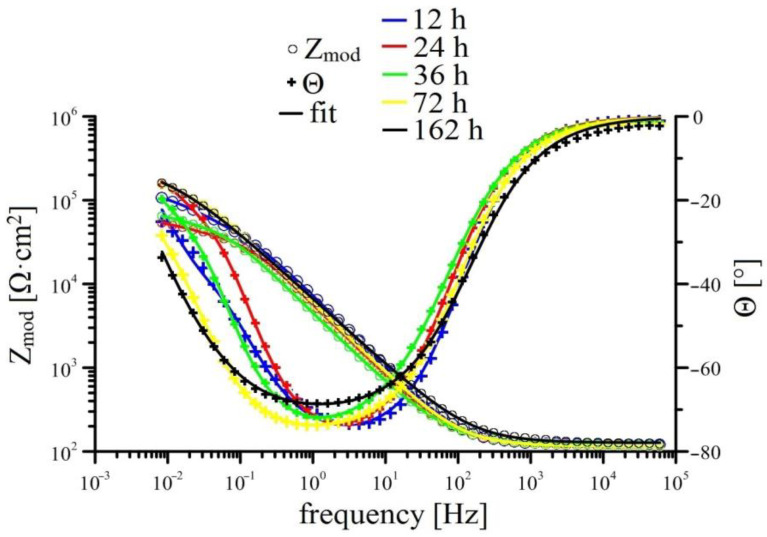
Time-dependent impedance spectra for Mn^(2)^ solution (Bode plot: log Z_mod_ vs. log frequency and phase angle vs. log frequency).

**Figure 11:**
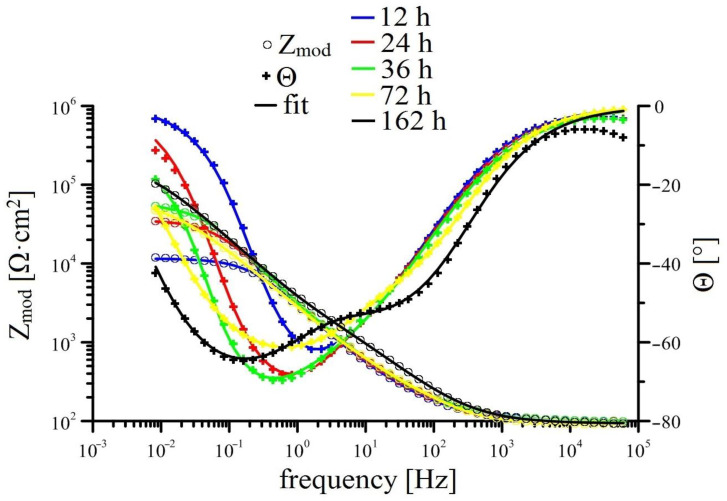
Time-dependent impedance spectra for Mn^(3)^ solution (Bode plot: log Zmod vs. log frequency and phase angle vs. log frequency).

**Figure 12:**
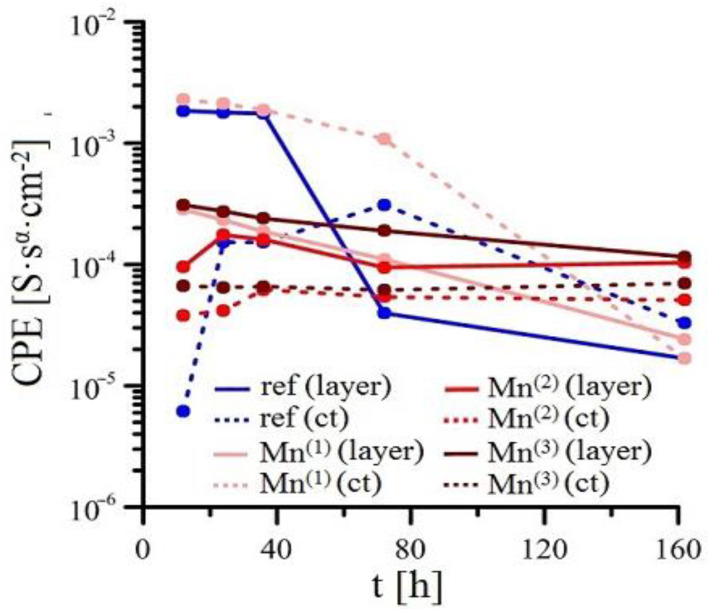
Point evaluation of CPE (t) EIS spectra for simulated concrete pore solution with graded amount of MnO_4_^−^.

**Figure 13:**
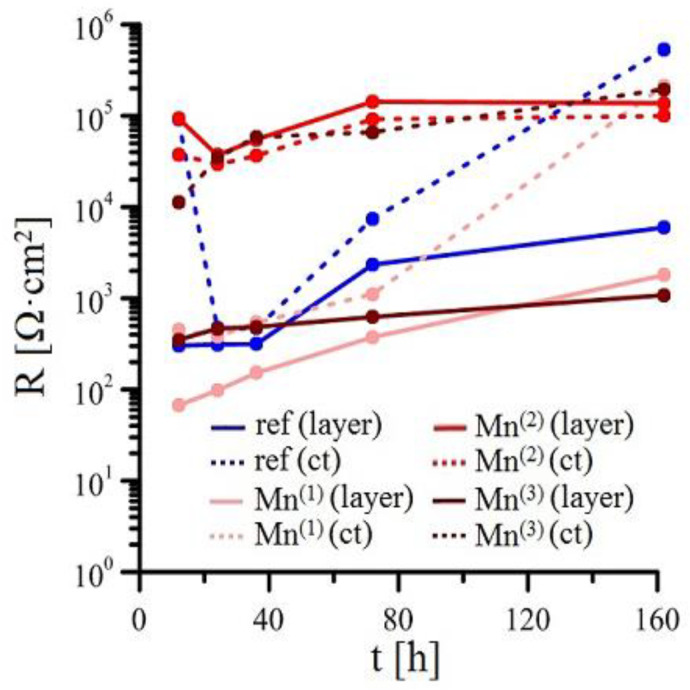
Point evaluation of R (t) EIS spectra for simulated concrete pore solution with graded amount of MnO_4_^−^.

Measurements of EIS for individual solutions is shown in Figure 8, Figure 9, Figure 10 and Figure 11 (Bode plot). Point evaluation (12 h; 24 h; 36 h; 72 h; 162 h) of CPE (t) is then in Figure 12, and R (t) in Figure 13.

In case of HDG exposure in simulated concrete pore solution without KMnO_4_, a rapid drop in R ref (ct) is observed initially, suggesting oxidized surface of HDG (higher content of ZnO/Zn(OH)_2_ [21,63,80]); after 24 h, a layer of corrosion products is formed (R ref (layer) increases). Between 36 h and 72 h R ref (layer) strongly increases and CPE ref (layer) drops, suggesting very intensive precipitation and growth of layer of corrosion products (CHZ; ZnO/Zn(OH)_2_). In this time interval, the thickness of the layer of corrosion products increases swiftly. Time dependence of intensification of growth corresponds to previously published results [14,41,81,82,83,84]. Simultaneously, the R ref (ct) increases, suggesting reduction of corrosion rate due to formation of corrosion products layer with barrier effect. Further increase of R ref (layer) after 72 h exposure is milder and can be explained by substantially slower growth of the layer compared to the time interval between 36 and 72 h. The growth of the layer is normally halted within 3 days of exposure [9,21,41].

In the case of Mn^(1)^ exposure, the EIS curve and fitted parameters are very similar to those measured in solution without MnO_4_^−^ however it is apparent that transition of HDG to passive state (formation of corrosion products with barrier properties) is delayed. R Mn^(1)^ (layer) increases steadily, a significant increase of R Mn^(1)^ (ct) is apparent only after 70 h of exposure. This corresponds to abrupt decrease of CPE Mn^(1)^ (ct) after 70 h, this again corresponds to increase of thickness of corrosion product layer but also shows delay in providing sufficient protection compared to reference. It is necessary to note that the value of R Mn^(1)^ (layer) at the end of exposure is lower than the same value in reference exposure. Therefore, it is apparent that corrosion products rich in Mn have in this case a negative effect on the overall barrier properties of precipitated mixture of corrosion products. It can be argued if competing cathodic reactions such as reduction of MnO_2_ to Mn (e.g., according to Equations (5) and (6), MnO_2_ formed by reduction of MnO_4_ (Equation (4)) do not limit the formation of common corrosion products based on (eventually ZnO/Zn(OH)_2_) to which the good protective capabilities up to pH 13.3 are normally ascribed to [39,74,75,81,82]. Inhibition of reaction (2) can correspond to partial mitigation of precipitation of corrosion products based on CHZ (ZnO/Zn(OH)_2_), limiting local increases in pH. Local increase in pH can be relevant to the precipitation of some coating components (limited solubility of alkaline forms of oxides, etc.) [93,94,95].

Exposure in Mn^(2)^ is characterized by high values of R Mn^(2)^ (layer) since the start of exposure; these values remain very stable. Similarly stable is the R Mn^(2)^ (ct). Significant changes are not apparent in either CPEct and CPElayer (see Figure 12). It is obvious that since the start of the exposure, a stable protective layer is formed, also strongly preventing RDHE (similar to [50]). Because the protective layer in reference exposure is formed only after 40 h, it is obvious that lower corrosion rates and corrosion of hot-dip galvanized coating without hydrogen evolution (Equation (2)) is caused by the presence of sufficient amounts of KMnO_4_. The effect of MnO_4_^−^ on corrosion behaviour of HDG in simulated concrete pore solution at pH 12.8 can be seen not only on type of cathodic reaction (probably even process of anodic corrosion reaction [50]) but also on the composition of corrosion products. Compared the EIS data from Mn^(1)^ exposure, R Mn^(2)^ (layer) is one order of magnitude higher at the end of exposure compared to reference exposure. Although the R_p_ values (see Figure 5) do not directly match, it can be argued that the composition of corrosion products on the surface of HDG (presence of Mn rich phases) in this case ensures at least comparable barrier properties as in reference exposure.

Last exposure of HDG in simulated concrete pore solution with highest amounts of MnO_4_^−^ (Mn^(3)^) exhibited relatively low R Mn^(3)^ (layer), comparable with values measured in Mn^(1)^ exposure. Simultaneously, high values of CPE Mn^(3)^ (layer) were recorded. This suggests precipitation of corrosion products with lower protective capabilities. However it is likely that corrosion product precipitate consists of a phase very rich in manganese [50], while the formation of zinc-based phase [75,82] will be most likely strongly suppressed. Stability of parameters discussed above during the whole exposure imply that protective layer is not formed, with restructuring of the layer of corrosion products being the likely cause. From Figure 13, the R Mn^(3)^ (ct) increases at the beginning of exposure and generally remains high. It is indicated that lower corrosion rates of HDG samples in this environment is caused by slow charge exchange (not by formation of sufficiently protective layer of corrosion products). It is apparent that in this case the corrosion mechanism of HDG can be different, similarly to exposure in Mn^(2)^ without hydrogen evolution. The cathodic reaction of hydrogen forming from water (reaction (2)) is here replaced by reduction of MnO_4_^−^, most likely occurring by subsequent reactions discussed earlier (Equations (4)–(7)). It can be argued that at the beginning of the (Mn^(3)^) exposure, the cathodic reaction of dissolved oxygen (Equation (8)) takes place and is later overtaken by reduction of MnO_4_^−^ in series of further reduction reactions of Mn [86,87,88].

It is clear that sufficient additions of KMnO_4_ to real concrete mixes can significantly reduce the corrosion of hot-dip galvanized reinforcement. However, it is important to note that other inorganic inhibitors also show significant corrosion suppression of HDG coatings in fresh and hardened concrete at sufficient additions to concrete. From this point of view, nitrite additions are mainly discussed [96,97], but according to the results of some papers, amines are more effective corrosion inhibitors [98,99]. Previously, plant extracted inhibitors have also been tested with positive inhibitory effects. Against localized corrosion damage induced by chloride anions, sodium eperuate from Wallaba wood extract was tested as an effective corrosion inhibitor of zinc in alkaline environment [100]. According to the results of other papers, *Lawsonia* extract [101], *Hibiscus subdariffa* extract [102] and also natural saccharides [103] have suitable inhibitory properties against zinc corrosion in alkaline environment.

In the case of the use of MnO_4_^−^ in reinforced concrete structures (HDG coating on concrete reinforcement), it is also necessary to verify the effect of the behaviour of this anion in case of corrosion of the coating in the presence of supercritical amounts of chloride anions. Chloride anions from the de-icing salts (NaCl, CaCl_2_) cause localized corrosion damage to the coating [4,76]. It is necessary to verify the inhibitory effect of this substance also in this case. The obtained data may extend the purposeful use of MnO_4_^−^ anions in the case of hot-dip galvanized concrete reinforcement. The inhibitory effectiveness of conventional corrosion inhibitors of conventional concrete reinforcement against the action of chloride anions is standardly reported [104,105,106,107,108].

### 3.3. Analysis of Corrosion Products

This chapter evaluates the composition of corrosion products on HDG in individual exposures. Their composition was studied using SEM (surface and cross sections of coating and corrosion products), X-ray diffraction, XPS analysis of top layer of corrosion products and comparison of content of calcium and manganese in the whole layer using AAS. For composition of HDG corrosion products study, separate sets of HDG samples. The samples were analyzed after 3 months of exposure in individual simulated concrete pore solutions.

#### 3.3.1. SEM Analysis

Scanning electron microscopy was used to evaluate morphology of corrosion products precipitates and also to produce elemental maps on cross sections (distribution of Zn/Ca/Mn/O).

**Figure 14:**
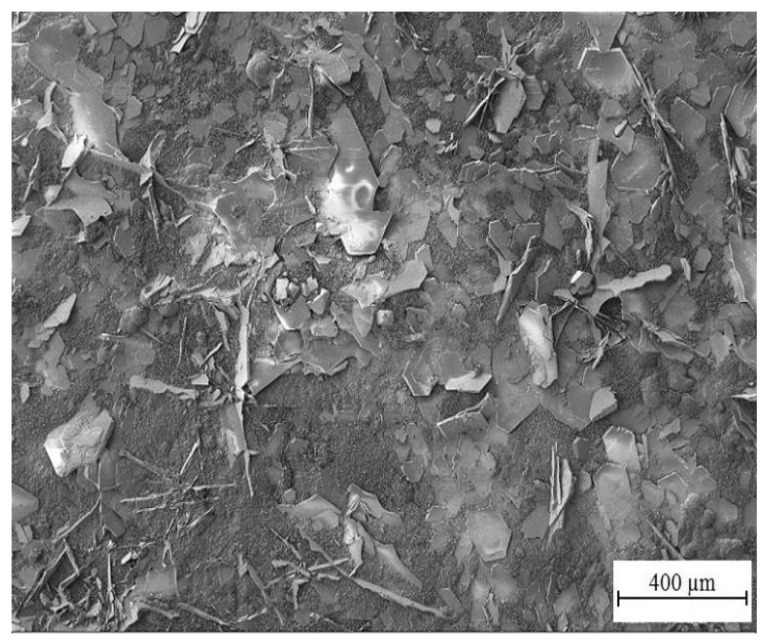
Morphology of precipitated corrosion products on HDG after 3 months’ exposure in simulated concrete pore solution of pH 12.8 without KMnO_4_.

**Figure 15:**
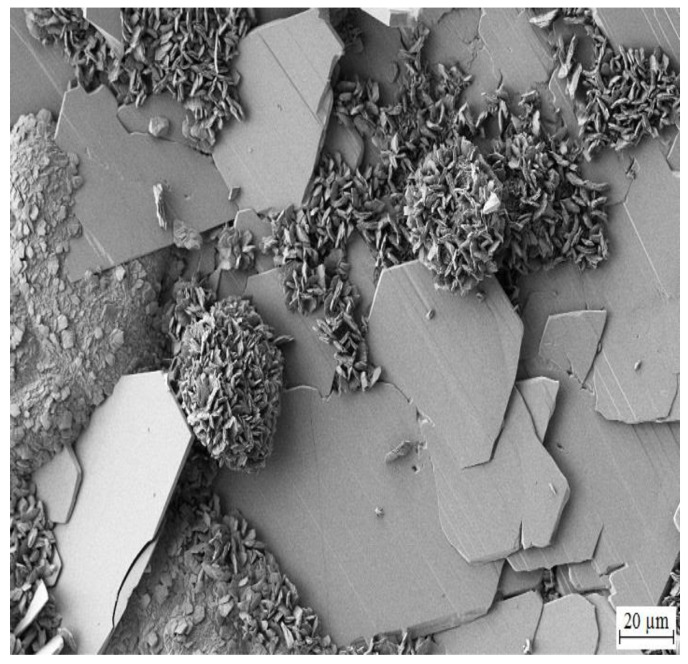
Detail view of corrosion products shown in Figure 14.

Figure 14 and Figure 15 show the morphology of corrosion products from simulated concrete pore solution without KMnO_4_. The surface is covered by crude fraction and also fine platelets (precipitated both in vertical positions and parallely to HDG surface) which are most likely composed of CHZ. These were observed and described in detail by numerous authors [2,9,14,81,82,109,110,111,112]. The ratio of surface coverage by platelets is high; no pores or unoccupied areas were apparent. Besides platelets, no other crystalline features were detected.

**Figure 16:**
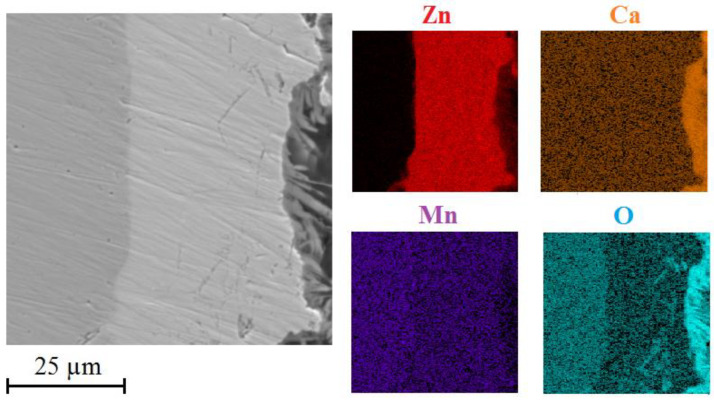
SEM—distribution of Zn/Ca/Mn/O on cross section in precipitate of corrosion products (exposure in simulated concrete pore solution of pH 12.8 without MnO_4_^−^ addition).

Figure 16 shows the distribution of selected elements (Zn/Ca/Mn/O) on cross section of HDG coating with top layer of corrosion products.

It is obvious that the corrosion products comprise uniformly distributed calcium, oxygen and zinc. Manganese is present all across the cross cuts. The palisade character of the corrosion products is in this case confirmed by the presence of oxygen. It can be discussed that the top layer of corrosion products includes oxygen but lacks zinc. This would indicate local precipitation of CaCO_3_ and Ca(OH)_2_ in the top layer of corrosion products which has also been proved earlier [113,114,115].

**Figure 17:**
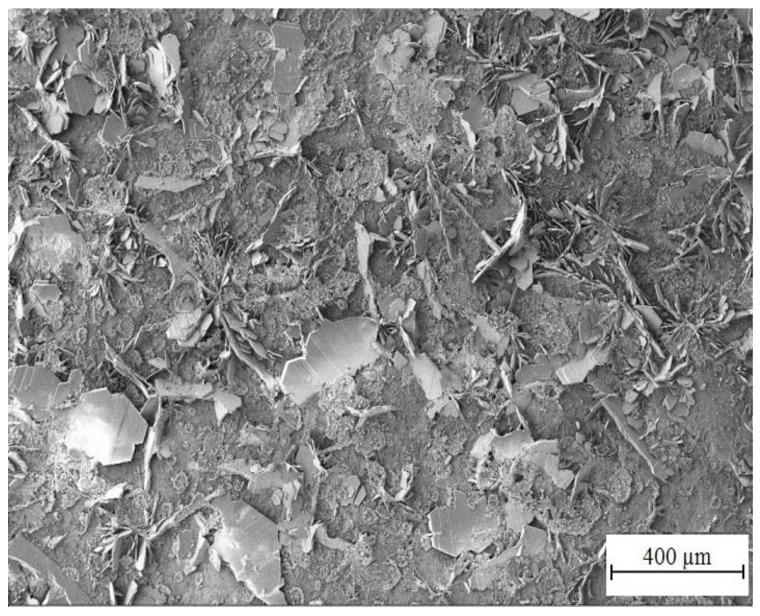
Morphology of precipitated corrosion products on the surface of HDG sample after exposure (3 months) in model concrete pore solution of pH 12.8 with addition of KMnO_4_^−^ (solution Mn^(1)^).

**Figure 18:**
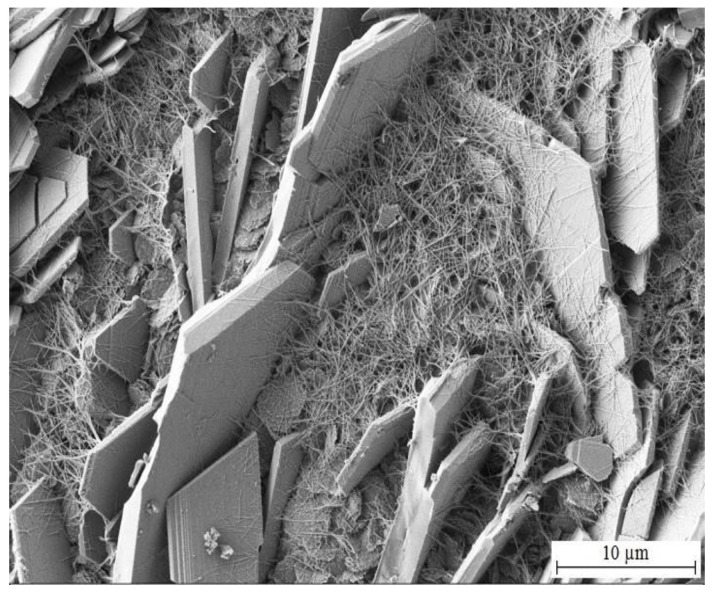
Detail view of precipitated corrosion products shown in Figure 17.

**Figure 19:**
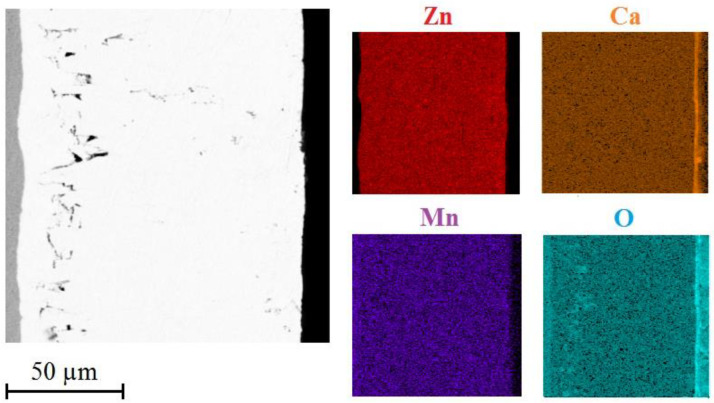
SEM—distribution of Zn/Ca/Mn/O on cross section in precipitate of corrosion products (exposure in simulated concrete pore solution of pH 12.8 with addition of KMnO_4_ (Mn^(1)^)).

The morphology of corrosion products precipitated on HDG samples after exposure in Mn^(1)^ solution is summarized in Figure 17 and Figure 18. Elemental maps from the exposure are shown in Figure 19. The surface is again covered by platelets (these are crystals CHZ), their orientation is in this case palisade-like and between these crystallites (nominaly lower number of platelets compared to reference exposure—Figure 14) there are thin fibers of phases which are not CHZ or common zinc corrosion products (ZnO/Zn(OH)_2_). Most likely these are amorphous phases of manganese oxo-hydroxides.

Compared with reference exposure, the thickness of the layer of corrosion products is lower (Figure 19). Presence of manganese in corrosion products is (in regard to scanned surface under corrosion products (see Figure 16)) is already probable.

The presence of zinc, calcium and oxygen is at expected levels and comparable to precipitate forming in simulated concrete pore solution without KMnO_4_.

**Figure 20:**
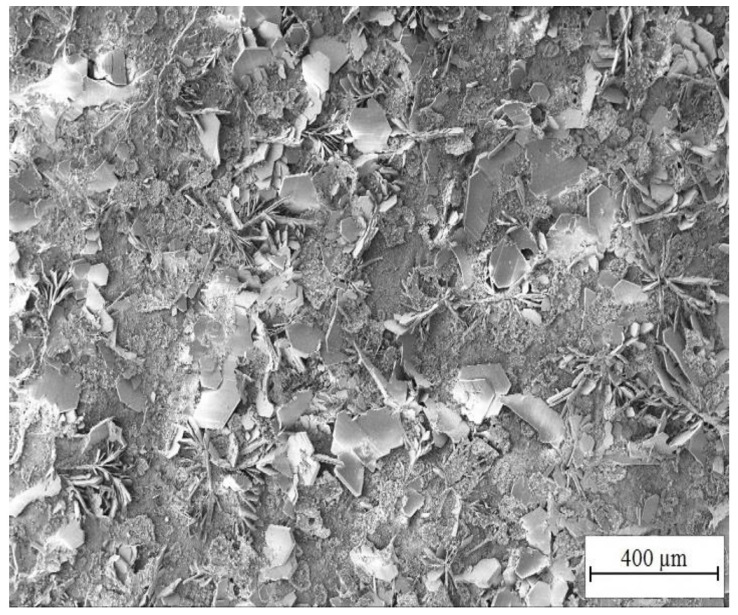
Morphology of precipitated corrosion products on the surface of HDG sample after exposure (3 months) in simulated concrete pore solution of pH 12.8 with addition of KMnO_4_^−^ (solution Mn^(2)^).

**Figure 21:**
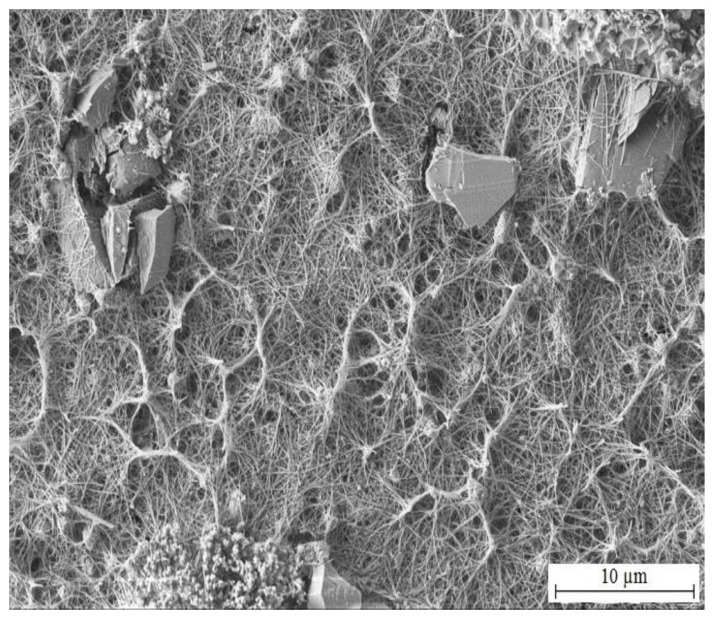
Detail view of precipitated corrosion products shown in Figure 20.

While the concentration of MnO_4_^−^ in simulated concrete pore solution Mn^(2)^ is already significant (10^−3^ mol·L^−1^), formation of CHZ (see Figure 20 and Figure 21) and apparently also ZnO/Zn(OH)_2_ is not completely suppressed. Platelets of CHZ are, in limited numbers, still formed, as well as a fiber-like phase, very densely filling the space between platelets. These are most likely Mn-rich phases (oxo-hydroxides).

**Figure 22:**
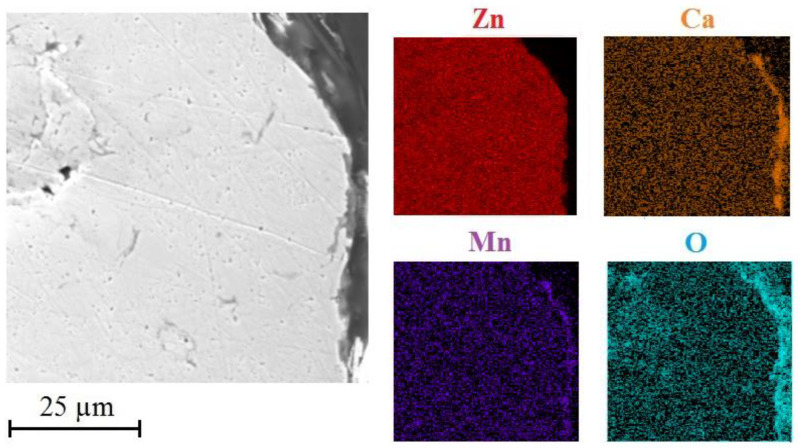
SEM—distribution of Zn/Ca/Mn/O on cross section in precipitate of corrosion products (exposure in simulated concrete pore solution with KMnO_4_ (Mn^(2)^)).

Figure 22 shows distribution of Zn/Ca/Mn/O through the layer of corrosion products after exposure in Mn^(2)^ solution. Compared to reference exposure, reduction of thickness of corrosion products precipitate is apparent. Presence of manganese in corrosion products is clear. Phases rich in manganese are also uniformly distributed across the whole thickness of the formed corrosion products. Distribution of zinc, calcium and manganese is expectable and does not show significant differences to reference exposure.

**Figure 23:**
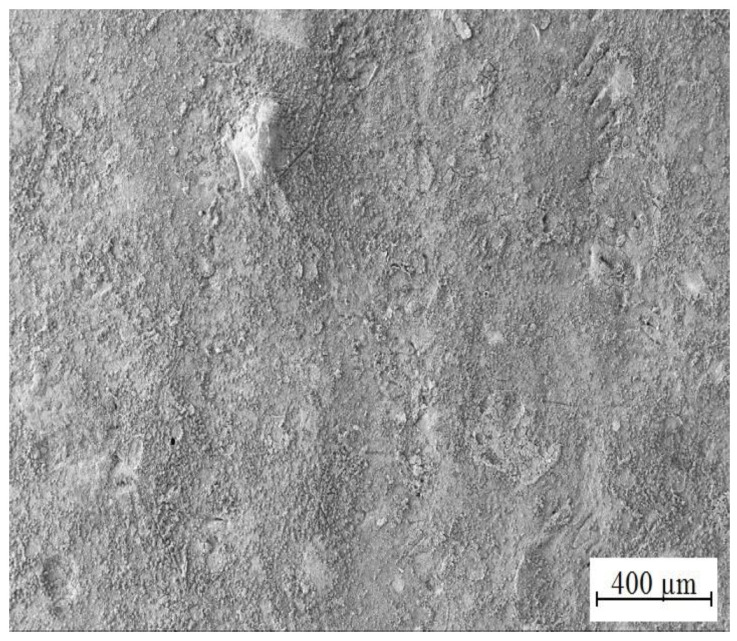
Morphology of precipitated corrosion products on the surface of HDG sample after exposure (3 months) in model concrete pore solution with addition of KMnO_4_^−^ (solution Mn^(3)^).

**Figure 24:**
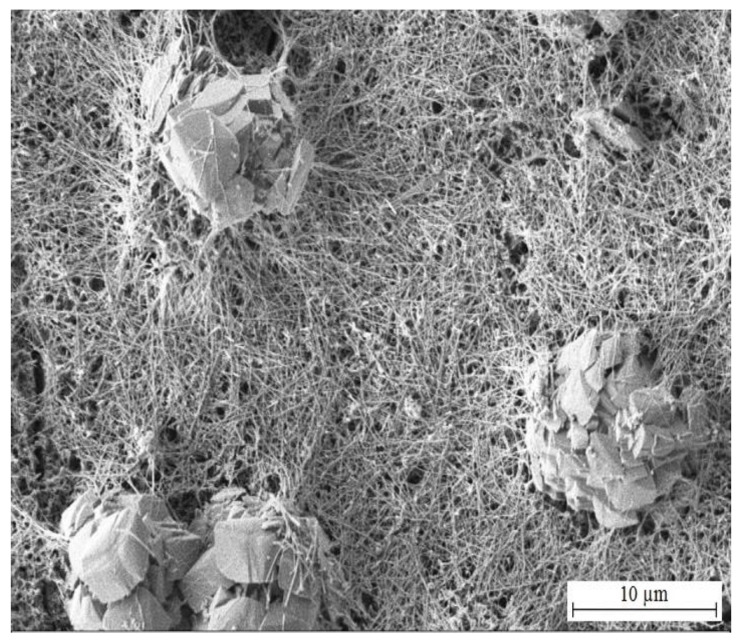
Detail view of precipitated corrosion products shown in Figure 23.

Highest chosen concentration of KMnO_4_ in simulated concrete pore solutions (Mn^(3)^) (10^−2^ mol·L^−1^) suppresses formation of CHZ platelets (see Figure 23 and Figure 24). It cannot be ruled out that formed clusters of atypical crystallites shown in Figure 24 are Ca[Zn(OH)_3_]_2_·2H_2_O (eventually ZnO/Zn(OH)_2_) however it is unlikely based on the morphology. Between these clusters, a fine network of very dense fibers, most likely with amorphous structure, is formed. The network is most dense in the precipitate (solution Mn^(3)^) and very similar to the texture of fiber visible in SEM images of hot-dip galvanized steel exposed in solutions Mn^(1)^ and Mn^(2)^. The formation of this precipitate is evidently related to the content of MnO_4_^−^ in electrolyte. Again, these are manganese-rich phases, probably a diverse mixture of amorphous oxo-hydroxides. Barrier properties of this network are most likely lower compared with the common layer of corrosion products forming on HDG zinc in simulated concrete pore solutions up to pH 13.3 (CHZ/ZnO/Zn(OH)_2_).

**Figure 25:**
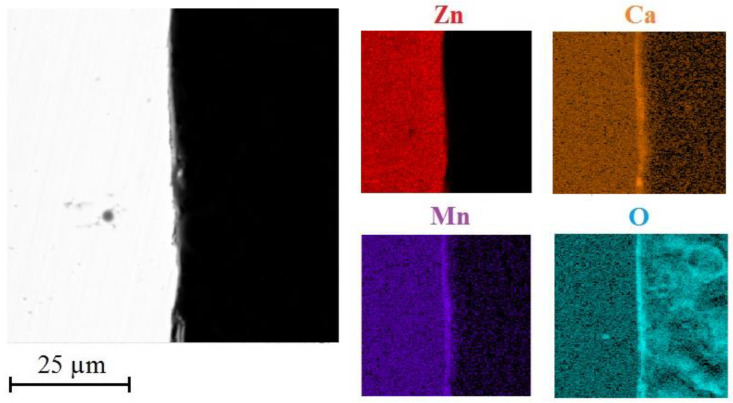
SEM—distribution of Zn/Ca/Mn/O on cross section in precipitate of corrosion products (exposure in simulated concrete pore solution with KMnO_4_ (Mn^(3)^)).

Figure 25 shows distribution of zinc, calcium, manganese and oxygen on a cross-section of a layer of corrosion products after exposure in solution Mn^(3)^. The images clearly show significant concentration of manganese in the whole layer of corrosion products. It is also apparent that these products contain significant amounts of calcium and oxygen. The formation of normal corrosion products cannot be in this environment ruled out.

#### 3.3.2. X-ray Diffraction Analysis

**Figure 26:**
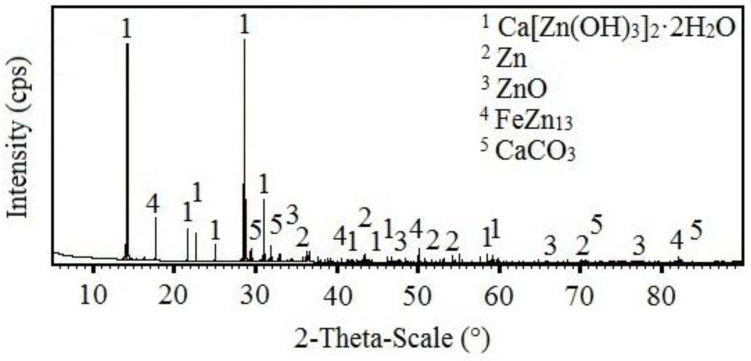
XRD analysis of HDG sample exposed for the duration of 3 months in simulated concrete pore solution of pH 12.8 without addition of MnO_4_^−^.

**Figure 27:**
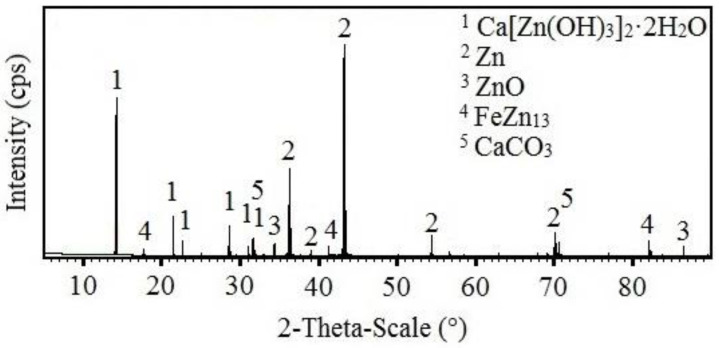
XRD analysis of HDG sample exposed for the duration of 3 months in simulated concrete pore solution of pH 12.8 with addition of MnO_4_^−^ (Mn(^1^)).

**Figure 28:**
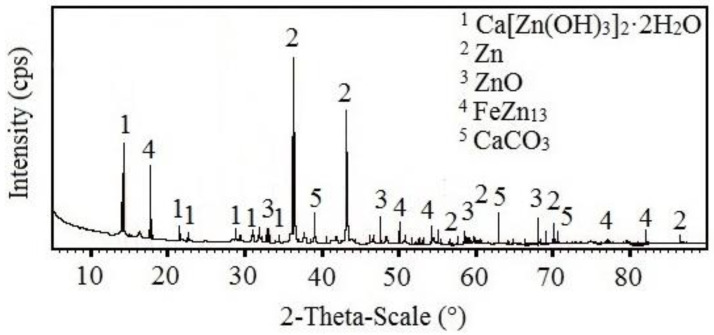
XRD analysis of HDG sample exposed for the duration of 3 months in simulated concrete pore solution of pH 12.8 with addition of MnO_4_^−^ (Mn(^2^)).

**Figure 29:**
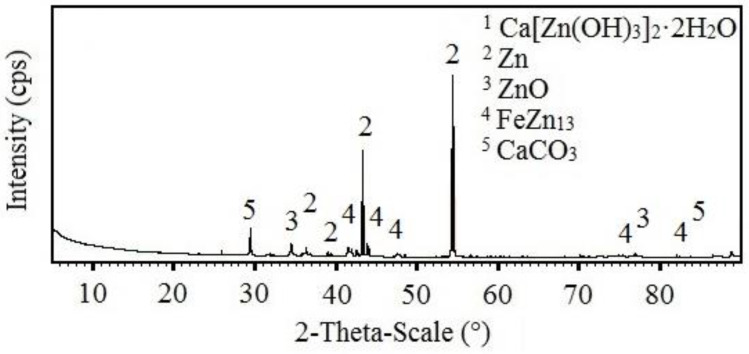
XRD analysis of HDG sample exposed for the duration of 3 months in simulated concrete pore solution of pH 12.8 with addition of MnO_4_^−^ (Mn(3)).

The results of the SEM analysis suggest that with increasing concentrations of KMnO_4_, ration of phases rich in manganese in corrosion products also increases; the expected phases are Mn_2_O_3_, MnO(OH), Mn_3_O_4_, MnO_2_ (see Figure 6). SEM cannot, however, distinguish the chemical structure of the phases with certainty. For this purpose, X-ray diffraction was implemented. The results from the analysis of corrosion products precipitate from reference exposure is shown in Figure 26, Mn^(1)^ in Figure 27, Mn^(2)^ in Figure 28, and finally, results from solution Mn^(3)^ are in Figure 29.

The reference sample clearly showed the presence of HDG corrosion products typical for exposure in simulated concrete pore solution up to pH = 13.3, i.e., Ca[Zn(OH)_3_]_2_·2H_2_O (CHZ) and ZnO [2,9,14,81,82,113,114,115,116]. The presence of CaCO_3_ in corrosion products was discussed in the previous sections. The present corrosion products still allow for the identification of substrate η phase (Zn) [63,64,116] and even phase deeper—ζ (FeZn_13_) [63,73,74,117,118].

With increasing concentrations of MnO_4_^−^, the amount of Ca[Zn(OH)_3_]_2_·2H_2_O decreases. After exposure in Mn^(3)^, CHZ was not detected at all. The analysis also showed the presence of ZnO in corrosion products (not the Zn(OH)_2_), even after exposure in solution with highest concentration of MnO_4_^−^. Obviously, Mn-based corrosion products (oxides/hydroxides/alkaline oxides) are amorphous [119,120,121,122] and therefore cannot be detected using X-ray diffraction.

#### 3.3.3. XPS Analysis

**Figure 30:**
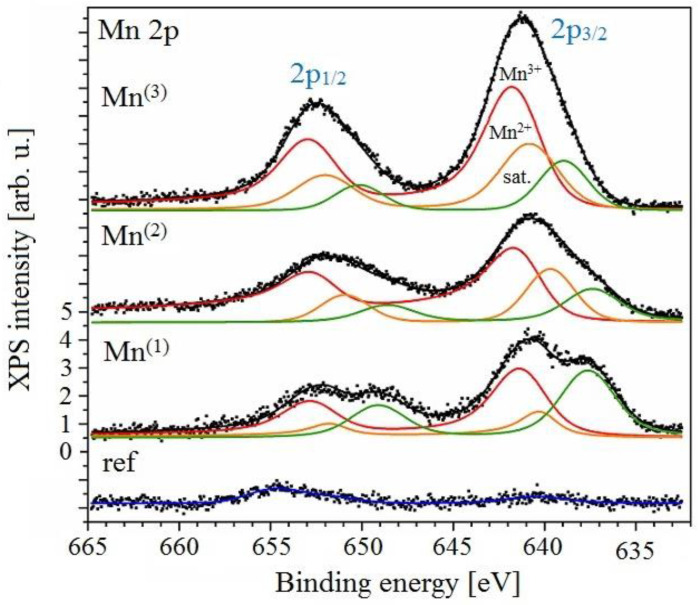
XPS spectrum of spectral region Mn 2p from all exposure—background subtracted from all spectra.

**Figure 31:**
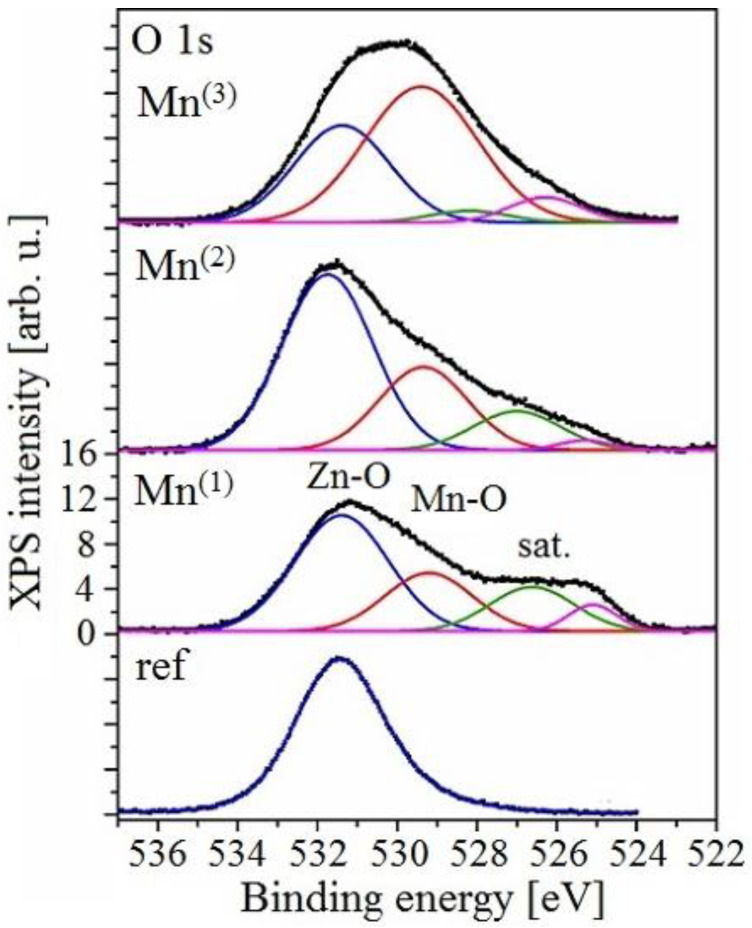
XPS spectrum of spectral region O 1s from all exposure—background subtracted from all spectra.

In Figure 30 and Figure 31, photoelectron spectra of Mn 2p and O 1s, respectively, are shown. Mn 2p doublets were fitted with asymmetric components (due to multiplet splitting), yielding a spin-orbit split 11.4 eV. In all cases, manganese was present in an oxidized form in various oxidation states. Particularly, Mn^2+^ and Mn^3+^ cations were found with peak positions at 640.3 eV and 641.2 eV [123,124], with the latter being the dominant contribution. No discernible amounts of tetravalent Mn^4+^ were detected. Mn 3s overlaps with strong Zn 3p peak (spectra not shown), however the spectral decomposition leads to results consistent with the Mn 2p line analysis, yielding a major contribution from trivalent Mn^3+^. Finally, this conclusion is also supported by the values of the Mn Auger parameter [125] calculated from positions of Mn 2p photoelectron peak and Mn L3M23M45 Auger transition. Thus, manganese is likely present in the form of MnO, Mn_2_O_3_, and Mn_3_O_4_ oxide phases and, eventually, MnOOH oxohydroxide.

In O 1s spectra (Figure 31), contributions from both zinc and manganese oxides are seen at 531.4 and 529.2 eV, respectively. The Zn-related peak is slightly from a position of ZnO at 531.3 eV, probably due to the non-stoichiometric and disordered nature of the oxide containing many oxygen vacancies and hydroxyl groups. It also shows a slightly increasing contribution of MnOx-related signal in the overall line intensity with increasing concentration (especially for 10 g), consistently with the behavior of the Mn 2p spectra.

It should be noted that the occurrences of the “satellite” peaks observed in most spectral regions at lower binding energies is a result of electronic band bending due to ferroelectric polarization [126], since ZnO is a wide-band gap semiconductor of native n-type due to doping by oxygen vacancies or zinc interstitials and all the samples were magnetized. The contribution of this component decreases with increasing concentrations of simulated concrete pore solution.

#### 3.3.4. AAS Analysis

**Figure 32:**
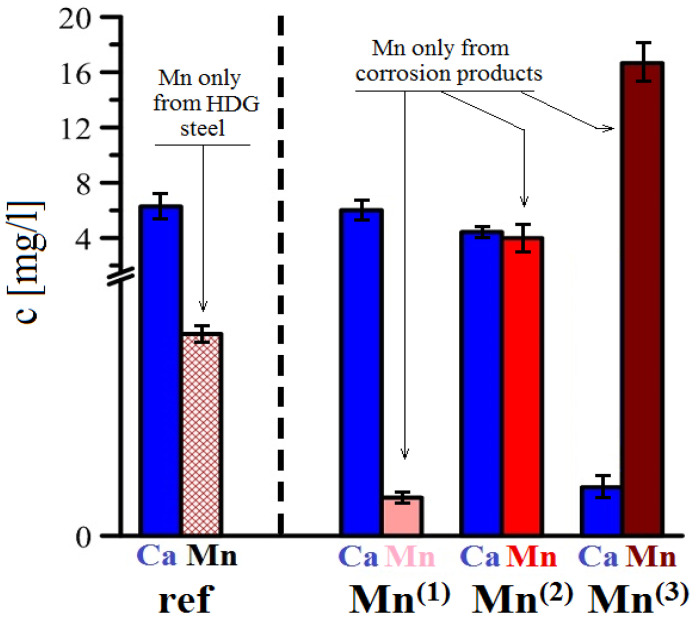
Resulst of AAS analysis comparing the content of Ca/Mn in corrosion products after pickling in HCl.

Corrosion products from HDG samples from individual exposure were removed using a solution of hydrochloric acid, and the contents of calcium and manganese in the solution was analyzed. The results for all simulated concrete pore solutions are shown in the bar chart in Figure 32. Reference solution (without addition of KMnO_4_), showed manganese concentration of approximately 1.2 mg/L. The source of manganese is obviously the dissolution of HDG steel [116,117,127]. This value was considered to the background and was subtracted from other values recorded in solutions with added MnO_4_^−^. The reason was to acquire more relevant data about Mn only in corrosion products. Cross comparison of Mn and Ca content in corrosion products show a simple trend. Increasing the MnO_4_^−^ concentration in simulated concrete pore solutions results in reduced content of Ca and increased content of Mn. After exposure in Mn^(2)^, the corrosion products show comparable amounts of manganese and calcium, however after exposure in Mn^(3)^ the amount of manganese is 10-fold higher compared with calcium. When X-ray results are taken into account, it is apparent that this value comes primarily from precipitated CaCO_3_ and not the Ca[Zn(OH)_3_]_2_·2H_2_O. According to Figure 32, the KMnO_4_ in concentration of 10^−4^ mol·L^−1^ (Mn^(1)^) does not result in precipitation of phases with significant content of manganese.

The highest concentration of MnO_4_^−^ is typical for the formation of less-protective corrosion products and fundamental changes in the corrosion mechanism of HDG.

The possible use of KMnO_4_ as a corrosion inhibitor of hot-dip galvanized reinforcement (preventing coating corrosion under the development of hydrogen and at the same time limiting anodic corrosion reaction) cannot be carried out without the clarification of the influence of MnO_4_^−^ anions on the hydration of cement and resistance of the resulting concretes to climatic influences (chloride anions, frost). It is clear from the literature that the contamination of developed concretes with metal oxides such as ZnO [128,129,130], PbO [131,132], or CuO [133,134] can significantly retard the hydration of conventional silicate cements. The contamination of concrete mixtures with sulphate anions can significantly limit the service life of the resulting concrete (gradual formation of so-called secondary ettringite–sulphate corrosion) [135,136,137,138]. Detailed conclusions of scientific works are not yet available on the influence of MnO_4_^−^ anions on the hydration reaction of convective silicate cements (and the durability of the formed concretes). The effects of these anions on the hydration, mechanical properties and durability of the concretes thus formed has not been directly investigated so far. The presence of MnO_4_^−^ in porous cement slurry foamed by H_2_O_2_ did not lead to abnormal hydration of Porland cement [51]. It was found that small additions of KMnO_4_ as a binding material to green concrete (recycled concrete with tyre rubber powder) led to an increase in concrete strength and durability [139]. Notwithstanding the above, it can definitely be summarized that MnO_4_^−^ cannot negatively affect the hydration of silicate cements, the mechanical properties of concrete or its durability. It is necessary to conduct experiments that directly observe the effect of these anions on these properties of concretes that are essential for practice.

## 4. Conclusions

Based on exposures in simulated concrete pore solutions of pH 12.8 with added (10^−4^; 10^−3^ a 10^−2^ mol·L^−1^), it can be confirmed that MnO_4_^−^ ions (10^−3^ mol·L^−1^) can be very effective in preventing hydrogen evolution during corrosion of hot-dip galvanized steel in concrete. The cathodic reaction is in this case the reduction of compounds with manganese in higher oxidation states, most likely Mn^IV^ to oxidation states Mn^II^/Mn^III^. These compounds rich in manganese are strictly amorphous and with increasing the concentration of MnO_4_^−^ in solution, the amount of Mn^III^ phases also increases. Further, with increasing amounts of KMnO_4_ in simulated concrete pore solutions, formation of calcium hydroxyzincate (CHZ) is suppressed. At 10^−2^ mol·L^−1^ MnO_4_^−^, formation of CHZ is prevented completely. This corresponds to relatively low protective (barrier) effect of formed corrosion products compared to reference exposure (corrosion product composition: Ca[Zn(OH)_3_]_2_·2H_2_O, ZnO/Zn(OH)_2_). Contrarily, at lowest MnO_4_^−^ concentrations (10^−4^ mol·L^−1^) there are no significant changes in the corrosion mechanism of hot-dip galvanized steel, that is the corrosion occurs with hydrogen evolution and the anodic process is uninhibited. At this concentration, normal formation of the zinc corrosion product layer (CHZ, ZnO/Zn(OH)_2_) is hindered and the time period of HDG corrosion with hydrogen evolution is prolonged. At the highest MnO_4_^−^ concentration, a significant reduction of corrosion takes place due to the inhibition of the charge transfer reaction (without hydrogen evolution). Nevertheless, a low-protective layer of corrosion products is formed with minimal content of zinc compounds.

The effective use of KMnO_4_ for real concrete mixtures with goal of reduction of hot-dip galvanized rebar corrosion with hydrogen evolution is very problematic since it is obvious that effective concentration range is narrow (optimally ~ 10^−3^ mol·L^−1^). Lower concentrations can prolong the corrosion of HDG steel with a high rate of hydrogen evolution. While high concentrations of MnO_4_^−^ ensure low corrosion rate and prevent hydrogen evolution, they also result in the formation of less-protective layers of corrosion products. This phenomenon can have a negative effect on the protective capabilities of HDG steel against chloride anions. For these reasons, the use of KMnO_4_ in real conditions of large concrete constructions would require the strict observance of the optimal dosage of this substance into the concrete mixture and at the same time prolonging the time of its homogenization (thorough mixing). The application of this inhibitor to concrete mixes during the construction of structures using hot-dip galvanized reinforcement would probably not have been possible without thorough control operations of the composition of the individual concrete mixes.

For the practical use of MnO_4_^−^ ions for reinforced concrete structures with implemented hot-dip galvanized reinforcement, it is necessary to verify their inhibitory effect in case of concrete contamination by supercritical amounts of chloride anions from de-icing salts (NaCl, CaCl_2_).

Environmental aspects of KMnO_4_ addition to cement mixtures make the application very problematic, since it can be released into environment.

Further research must focus on the use of corrosion inhibitors with similar effects to those of KMnO_4_ but with a broader interval of effective concentration. These compounds also need to be environmentally-friendly and non-toxic to humans.

## Figures and Tables

**Table 1:** Manufacturer-guaranteed steel composition.

C	Al	Si	P	S	Cr	Mn	Cu	Ni	Fe
0.10–0.14	<0.1	0.18–0.22	<0.03	<0.03	0.1–0.3	0.40–1.20	0.25–0.45	<0.3	bal.

**Table 2:** Detail characterization of simulated concrete pore solution with KMnO_4_.

Solution Denomination	pH	Composition of Simulated Concrete Pore Solution
ref	12.8	saturated Ca(OH)_2_ + KOH
Mn^(1)^	12.8	saturated Ca(OH)_2_ + KOH + 10^−4^ mol·L^−1^ KMnO_4_
Mn^(2)^	12.8	saturated Ca(OH)_2_ + KOH + 10^−3^ mol·L^−1^ KMnO_4_
Mn^(3)^	12.8	saturated Ca(OH)_2_ + KOH + 10^−2^ mol·L^−1^ KMnO_4_

## Data Availability

Data is contained within the article.
